# UGM: a more stable procedure for large-scale multiple testing problems, new solutions to identify oncogene

**DOI:** 10.1186/s12976-019-0117-1

**Published:** 2019-12-23

**Authors:** Chengyou Liu, Leilei Zhou, Yuhe Wang, Shuchang Tian, Junlin Zhu, Hang Qin, Yong Ding, Hongbing Jiang

**Affiliations:** 1 Department of Medical Engineering, Nanjing First Hospital, Nanjing Medical University, Nanjing, China; 2 Department of Critical Care Medicine, Nanjing First Hospital, Nanjing Medical University, Nanjing, 210016 Jiangsu China; 3 Department of Mathematics and Computer, Nanjing Medical University, Nanjing, China; 4 Department of Biomedical Engineering, Nanjing Medical University, Nanjing, China; 5 Hongbing Jiang, Nanjing Health Information Center, Nanjing, 210016 Jiangsu China

**Keywords:** Differentially expressed genes, False discovery rate, Standard deviation, RNA-Seq data, Root mean square error, Cancer-associated genes

## Abstract

Variations of gene expression levels play an important role in tumors. There are numerous methods to identify differentially expressed genes in high-throughput sequencing. Several algorithms endeavor to identify distinctive genetic patterns susceptable to particular diseases. Although these processes have been proved successful, the probability that the number of non-differentially expressed genes measured by false discovery rate (FDR) has a large standard deviation, and the misidentification rate (type I error) grows rapidly when the number of genes to be detected become larger. In this study we developed a new method, Unit Gamma Measurement (UGM), accounting for multiple hypotheses test statistics distribution, which could reduce the dependency problem. Simulated expression profile data and breast cancer RNA-Seq data were utilized to testify the accuracy of UGM. The results show that the number of non-differentially expressed genes identified by the UGM is very close to the real-evidence data, and the UGM also has a smaller standard error, range, quartile range and RMS error. In addition, the UGM can be used to screen many breast cancer-associated genes, such as BRCA1, BRCA2, PTEN, BRIP1, etc., provides better accuracy, robustness and efficiency, the method of identification differentially expressed genes in high-throughput sequencing.

## Introduction

Cancer is a major public health problem worldwide. It is a disease that arises from uncontrolled cell cycle, proliferation and inter-cellular communication. As of to date, more than 100 types of cancers were diagnosed in human [1]. Scientists have reached a consensus that cancer is caused by both genetic factors, such as mutations and disrupted hormones, and environmental factors [2]. Some tumors are hereditary diseases, which are attributed by the disorder of the mechanism regulating cell growth and proliferation. In general, genetic or epigenetic changes in DNA could confer a normal cell potential malignancy [3, 4]. Cellular- oncogenes, anti-oncogene and DNA repair genes are major types of genes that contribute to this process. The interaction of these genes is sometimes referred to as the “driver” of cancer [5].

Although the genomic composition of cells are almost identical for an individual, genetic, transcriptional and expression variation may occur during cell differentiation and proliferation. Investigation into the difference of gene expression profiles among cells in different state would provide significant insights into the function of genes and their products [6]. The identification of affiliation/connection between disease and genetic or expressional pattern renders tremendous/enormous significance. Differentially expressed genes and proteins can be screened from the level of genes and proteins, respectively.. Screening differential molecules can be accomplished in two ways: screening from protein expression data or using RNA-Seq data to detect differentially expressed genes. Over the past decade many genome-wide studies have demonstrated that there are many genes harboring overrepresented mutations, such as tumour protein 53 (TP53) [7], phosphatase and tensin homolog deleted on chromosome ten (PTEN) [8], kirsten rat sarcoma viral oncogene homolog (KRAS) [9], myelocytomatosis viral oncogene (MYC) [10], breast cancer (BRCA) [11] .

Gene chip is also known as Bio-array or microarray, and this technology is based on the theory of hybridization by Edwin Mellor Southern. In the 1980s, gene chip prototype was proposed. The first gene chip was achieved in 1991. With the development of human genome project and molecular biology technology, gene chip technology has been developing rapidly in the past 20 years. Gene chip can detect the growth of tumor-related information, and has evolved to be a sophisticated technology in tumor detection and analysis. The rapid development of gene chip technology has brought revolutionary impact on medical research [12].

Genomics research shows that the gene expression differences are associated with biological conditions and disease stages. It is a useful tool of microarray technology for quantitative analysis of gene expression in recent decades. Both the microarray data and RNA-Seq data is characterized by low sample size and high dimensional variables. Therefore, when identifying differentially expressed genes in these data, multiple comparisons are required. When we conduct multi-sample hypothesis tests, the false discovery rate (FDR) is a widely adopted method to control type I errors in null hypothesis testing. The FDR method is a probability designed to control the false events [13, 14]. For type I error, the FDR controlling procedure is not as strict as family wise error rate (FWER) controlling procedures, which controls the probability of more than one type I error [15]. Therefore, FDR controlling programs have an advantage over type I errors, but at the cost of increasing the error rate [16, 17]. At the same time, the results of different methods are quite different. So far, there is still no unified conclusion in the scientific community regarding the most efficient, robust and accurate method. Therefore, this paper aims to propose a new method for screening differentially expressed genes based on gene expression profiling data, and uses simulated gene chip data and breast cancer data to verify the validity and accuracy of the proposed method. Furthermore, this article also aims to provide a case study for the screening of clinical differentially expressed genes.

## Methods

### Multiple hypothesis testing and FDR

In the 1950s, multiple hypothesis testing began to gain attraction, especially for high-throughput data analysis, where the problem of multiple comparisons was particularly outstanding. Microarray data is an example of high-dimensional data, which is characterized by small sample size and high variable dimension, which constituted a typical multiple hypothesis testing problem. Table 1 summarizes this situation in traditional form.

1 $$ FDR=\left\{\begin{array}{c}E\left(\frac{V}{S+V}\right)=E\left(\frac{V}{R}\right)\ R\ne 0\\ {}0\ R=0\end{array}\right. $$

**Table 1:** Multiple hypothesis testing

	Declared non-significant	Declared significant	Total
True null hypotheses	U	V	m_0_
Non-true null hypotheses	T	S	m_1_(m-m_0_)
	m-R	R	M

We need to consider testing the m (null) hypothesis, where m0 is true and the rest m1 = m-m0 is false. After testing the m (null) hypotheses, there are R rejected and m-R not rejected null hypotheses. m (null) hypotheses were committed into four parts by type I error and type II error. They are U, V, S, and T. U and S denote the number of correct tests in m. V denotes the number of type I error tests in m. T represents the number of type II error in m, and R is used to represent observable random variables. U, V, S, and T are unobservable random variables

In the 1990s, Benjamini and Hochberg put forward the FDR control method. FDR control method uses correction theory to correct the first type of error in multiple hypothesis testing. In the rejected events, FDR controls the prospective rate of falsely rejected null events (type I errors) [15]. FDR is a relatively conservative comparison method, with greater power, compared with FWER control. FDR is outlined as follows

The definition of FDR is the expectation of false discovery rate(V/R). At present FDR has been widely used in practical problems. According to the literature reported, when *m*_0_ = *m*, then FDR = FWER. When *m*_0_ ≤ *m*, then *FDR* ≤ *FWER*. FDR not only improves the test capability, but also makes better the traditional multi-hypothesis test process, which is too conservative. Therefore, FDR supplies a applicable error calculation standard for multiple tests of large-scale data. FDR commonly used control process Benjamini, & LIU (BL), Benjamini, & Hochberg (BH), Benjamini & Yekutieli (BY) and a-daptive linear step- up (ALSU). Currently the most widely used method is the ALSU procedure. The ALSU procedure as follows:

1. Let *H*_01_, *H*_02_, *H*_03_, …, *H*_0*m*_ be the tested null hypotheses. Using single test method to test each event and get *P* values *P*_1_, *P*_2_, *P*_3_, …, *P*_*m*_, and sort *p* values ${P}_1^{\ast },{P}_2^{\ast },{P}_3^{\ast },\dots, {P}_m^{\ast }$.
2. Let $r\left(\lambda \right)=\underset{1\le i\le m}{\max}\left\{i:{P}_i^{\ast}\le \lambda \right\}$, where λ is usually taken as 0.5. *r*(*λ*) represents the number less than λ.
3. Estimate ${\hat{\pi}}_0$ by ${\hat{\pi}}_0=\frac{m-r\left(\lambda \right)}{m\ast \left(1-\lambda \right)}$. Estimate ${\hat{m}}_0$ by ${\hat{m}}_0=\frac{m-r\left(\lambda \right)}{1-\lambda }$, where ${\hat{m}}_0$ is the number of true vents.
4. Estimate $$ \hat{k}=\arg \underset{1\le i\le m}{\max}\left\{i:{P}_i^{\ast}\le \frac{i}{m}\ast \alpha \right\} $$. Where *α* = 0.05.
5. If $\hat{k}$ exists, reject the events of ${H}_{0(1)}^{\ast },{H}_{0(2)}^{\ast },{H}_{0(3)}^{\ast },\dots, {H}_{0\left(\hat{k}\right)}^{\ast }$. Else, do not reject any hypotheses.
6. Adjust $$ {P}_i^{\ast } $$ by $$ {P}_i^{\ast }=\underset{i\le k\le m}{\min}\left\{\min \left\{\frac{\hat{m_0}}{k}\ast {P}_k^{\ast },1\right\}\right\} $$.

From the above introduction, we can figure out that the key step of the ALSU procedure is the appraisal of m_0_. The accuracy of m_0_ is crucial for the screening of differentially expressed genes, FDR control processes and testing capabilities. However, statisticians found that this approach is very unstable [18]. In spite of the fact that we repeated many times FDR procedure and get the mean of m_0_ is exactly similar to the true value, the standard deviation (SD) is very large, which caused wide random deviation. Therefore, it is necessary to improve the estimation algorithm of m_0_.

### New estimation method

**Fig. 1:**
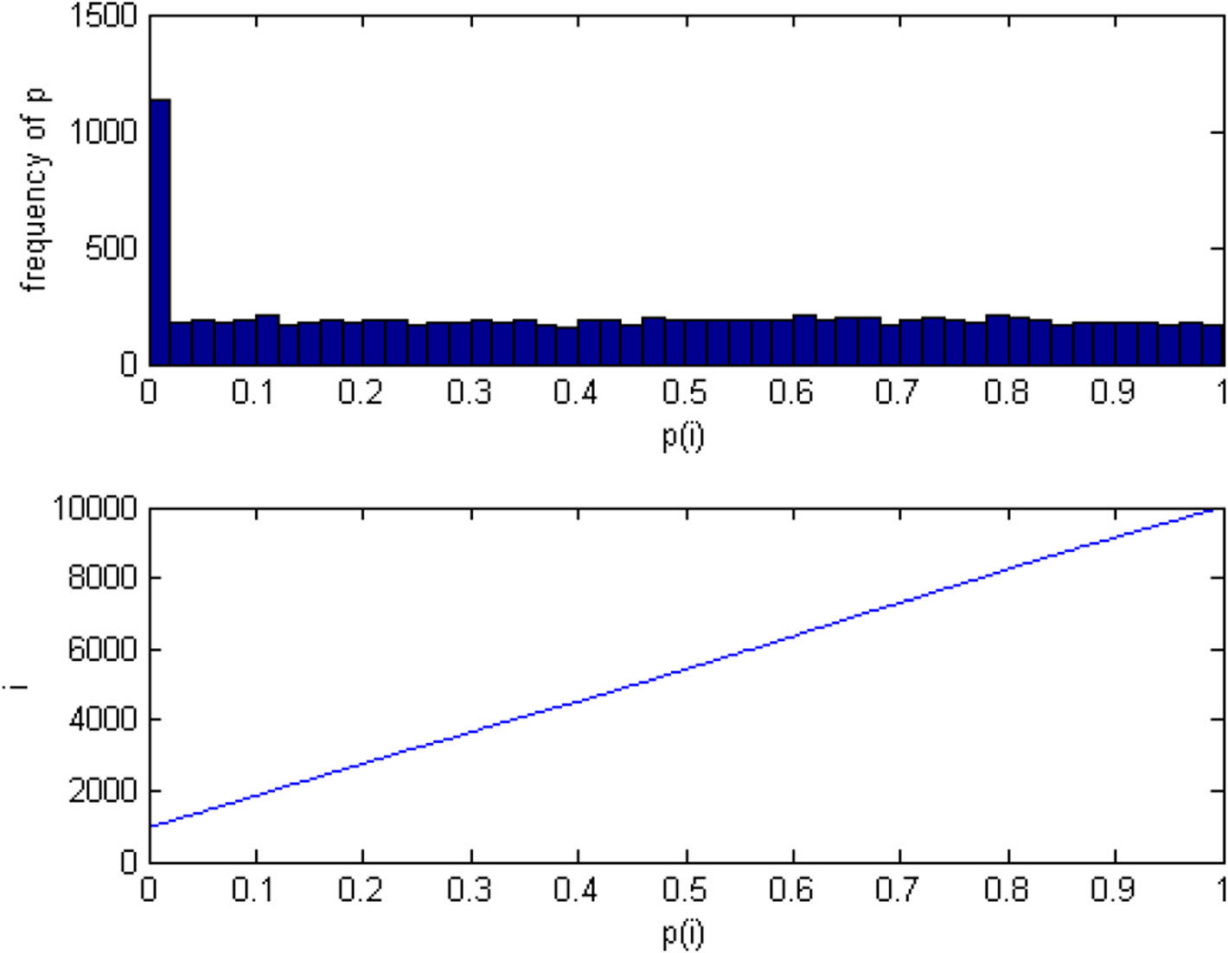
Relationship between P(i) and its frequency; P(i) and i are simu-lated data (10,000 genes). **a**. Frequency distribution of P(i); **b**. P(i) vs i. Note: Hypothesis testing has the following two characteristics. I). When H_0_ is true, the *P* value calculated from observed value is uniform distribution in (0, 1), i.e., P ~ U (0, 1). II). When H_0_ is false, the distribution of P value is uncertain. However according to the definition of P value can be known, this time P value is small. Usually the P value is less than 0.05, and tends to zero

The *P*-value is the probability that the sample emerge extreme results when the null event is true. In the hypothesis test, the P-value is used to determine the hypothesis test results and reflects the feasibility of the test results, i.e., the level of accepting and rejecting the null hypothesis. The smaller *P* value, the more significant the hypothesis test result. If we assume the null hypothesis is H_0_, the alternative hypothesis is H, and the sample observations are *X*_1_, *X*_2_, *X*_3_, …, *X*_*n*_. After selecting the appropriated statistic T, we can compute the corresponding *P* value. In multiple hypothesis tests, the *P*-value results are shown in Fig. 1.

From Fig. 1 we can get that P value is a very regular nature in the ideal state. If the number of genes is m, and the ratio of the number of non-differentiated genes is *π*_0_, therefore the number of non-differentiated genes are *m*_0_ = *m* ∗ *π*_0_. Assuming there is a value *γ*, which all differential expression of gene test *P* values are distributed in (0, *γ*). In this case, the genes distributed in (*γ*, 1) should be all non-differentially expressed genes. In this region, the number of non-differentially expressed genes in unit gamma length were $\underset{1\le i\le m}{\min}\left\{i:{P}_i^{\ast}\ge \gamma \right\}\ast \frac{\gamma }{1-\gamma}\#\left\{{\mathrm{P}}_{\mathrm{i}}\ge \upgamma \right\}\ast \frac{\upgamma}{1-\upgamma}$. Therefore the number of genes distributed in (0, *γ*) should theoretically be the sum of all the differentially expressed genes and $\underset{1\le i\le m}{\min}\left\{i:{P}_i^{\ast}\ge \gamma \right\}\ast \frac{\gamma }{1-\gamma }$, i.e., the number of genes in (0, *γ*) is $m-{m}_0+\underset{1\le i\le m}{\min}\left\{i:{P}_i^{\ast}\ge \gamma \right\}\ast \frac{\gamma }{1-\gamma}\mathrm{m}-{\mathrm{m}}_0+\frac{\upgamma}{1-\upgamma}\#\left\{{\mathrm{P}}_{\mathrm{i}}\ge \upgamma \right\}.$. In order to avoid the effect of random error, we calculated the number of non-differentially expressed genes in the multi-gammas.

The key of this algorithm is to appraise *m*_0_m_0_. Let *H*_01_, *H*_02_, *H*_03_, …, *H*_0*m*_ be null hypothesis (genes). Correspondingly, the *P*-values of independent hypothesis tests are *P*_1_, *P*_2_, *P*_3_, …, *P*_*m*_. Level of significance is α. Because this article uses the concept of the unit gamma length number of genes. In this paper, the algorithm is named Unit Gamma Measurement (UGM), which process as follows:

1. Let *H*_01_, *H*_02_, *H*_03_, …, *H*_0*m*_ be the tested null hypotheses. Using single test method to test each event and get *P* values *P*_1_, *P*_2_, *P*_3_, …, *P*_*m*_, and sort *p* values ${P}_1^{\ast },{P}_2^{\ast },{P}_3^{\ast },\dots, {P}_m^{\ast }$.
2. Select the appropriate cutoff gamma, which is used to qualitatively divide the P value. Gamma should be greater than the Level of significance. Gamma can be appropriately increased when there are lots of genes. Calculate the number of genes distributed in (0, *γ*), (*γ*, 2*γ*), …, (*n* ∗ *γ*, (*n* + 1) ∗ *γ*). (*n* + 2) ∗ *γ* was greater than 1. We define *Pre* _ *γ* and *Lat* _ *γ*(*k*) as follows:

2 $$ \left\{\begin{array}{c}\mathit{\Pr}{e}_{\gamma }=\underset{1\le i\le m}{\max}\left\{i:{P}_i^{\ast}\le \gamma \right\}\\ {} La{t}_{\gamma (k)}=\underset{1\le i\le m}{\max}\left\{i:{P}_i^{\ast}\le k\ast \gamma \right\}\ k=1,2,3,\dots, n\end{array}\right. $$

(3) Estimate *m* − *m*_0_. Estimation method as follow:

3 $$ m-{m}_0={\hat{m}}_1= Pre\_\gamma -\sum \limits_{i=1}^n{\tau}_i\ast Lat\_\gamma (i) $$

*τ*_*i*_τ_i_ was weight coefficient, which formula is as follows:

4 $$ {\tau}_i=\frac{1}{Lat\_\gamma (i)\ast {\sum}_{j=1}^n\frac{1}{Lat\_\gamma (i)}} $$

(4) Get $$ {\hat{m}}_0 $$

5 $$ {\hat{m}}_0=m-{\hat{m}}_1 $$

(5) Adjust $$ {P}_i^{\ast } $$ by $$ {P}_i^{\ast }=\underset{i\le k\le m}{\min}\left\{\min \left\{\frac{\hat{m_0}}{k}\ast {P}_k^{\ast },1\right\}\right\} $$.

### Simulation experiment and evaluation parameters

We use in silico analysis to generate gene expression profiles according to the data structure presented in Table 2. The sample size of the experimental group (patient group) and the control group (normal observation group) is 40. The population mean of gene expression levels of experimental group and control group is *μ*_1*i*_ and *μ*_2*i*_. When the gene (non-differentially expressed gene) number is less than m_0_, *μ*_1*i*_ = *μ*_2*i*_ = *μ*μ_1i_ = μ_2i_ = μ. When the gene (differentially expressed gene) number is more than m_0_–1, *μ*_1*i*_ ≠ *μ*_2*i*_. In order to avoid the impact of accidental factors on the results, we performed 1000 repeated experiments on the algorithm for different values of *π*_0_.

**Table 2:** Constitution of the gene expression profiles

Gene category	Gene number	Samples S1	Samples S2
		1 to 40	1 to 40
Non differentially expressed genes	Gene 1	X_11_~N(μ, 1)	X_12_~N(μ, 1)
	Gene 2		
	Gene m_0_		
Differentially expressed genes	Gene m_0_ + 1	X_21_~N(μ_1_, 1)	X_22_~N(μ_2_, 1)
	Gene m_0_ + 2		
	Gene m		

*μ*~*N*(0, 2), *μ*_1_~*N*(0, 1), *μ*_2_~*N*(2, 1). Non-differentially expressed genes’ number is from 1 to m0, which samples S1 and S2 come from a same population, i.e., *μ*_1*i*_ = *μ*_2*i*_ = *μ*μ_1i_ = μ_2i_ = μ. μ_1_ = μ_2_ = μ~N(0, 2); Differentially expressed genes’ number is from m_0_ + 1 to m, i.e., μ_1_ ≠ μ_2_, μ_1_~N(0, 1), μ_2_~N(0, 2)*μ*_1*i*_ ≠ *μ*_2*i*_

## Results

### Performance on simulated data

In general, the proportion of differentially expressed genes was small, i.e., *π*_0_π_0_ was large. In the simulation, we set the total number of genes (m) was 10,000, 8000, 5000, 3000, 2000 and 1000. We set the value of *π*_0_π_0_ was 0.8,0.85,0.9 and 0.95. In each case, we estimated the m_0_ using Adaptive Benjamini and Hochberg (ABH), Storey & Tibshirani-λ (S~λ), Two Stages Test (TST) and UGM methods and computed the average of m_0_ with repeated 1000 times simulations.

Table 3 showed the mean of m_0_ estimated by ABH, S-λ, TST, UGM in different conditions. We used the estimated m0 values and the actual m_0_ value to do the relative error analysis. The result shows that the relative error of the UGM method is distributed between − 0.181 and 0.156%. The relative error of the other three estimation methods were distributed between 0.071 and 5.900%, − 0.708 and 0.431%, − 4.873% and − 4.633%. The estimation results of m_0_ in the four methods have identical tendency as the actual value. However, the results of the UGM method and the ABH, TST method have significant difference (*P* = 0.01296, *P* = 0.0000, chi-square test), which is undetected between the UGM method and the S-λ method (*P* = 0.8644).

**Table 3:** Estimate the number of non - differentially expressed genes

M	m_0_	The conventional algorithm for estimating m0			UGM
		ABH	S~λ	TST	
m = 10,000	9500	9517.19	9508.02	9050.38	9506.46
	9000	9009.29	8994.82	8567.83	8998.81
	8500	8506.22	8501.08	8094.84	8499.64
	8000	8005.65	8016.6	7621.17	8002.45
m = 8000	7600	7618.56	7594.42	7238.49	7605.38
	7200	7212.48	7202.38	6857.02	7201.19
	6800	6806.34	6797.70	6476.21	6802.57
	6400	6405.70	6389.94	6092.90	6394.36
m = 5000	4750	4769.55	4747.70	4523.67	4746.36
	4500	4510.81	4494.24	4286.04	4501.43
	4250	4256.13	4248.34	4044.48	4247.84
	4000	4005.39	4001.74	3806.76	3999.08
m = 3000	2850	2873.10	2842.86	2712.85	2847.21
	2700	2712.24	2704.64	2571.58	2699.57
	2550	2557.97	2542.64	2431.85	2552.04
	2400	2405.60	2396.16	2286.61	2399.08
m = 2000	1900	1920.93	1907.90	1810.11	1900.54
	1800	1810.78	1804.56	1715.32	1802.80
	1700	1709.40	1707.18	1619.89	1700.99
	1600	1605.61	1599.80	1522.54	1597.70
m = 1000	950	972.36	949.10	904.86	949.81
	900	911.96	897.02	857.14	901.10
	850	856.03	843.98	809.41	849.86
	800	805.23	800.84	761.54	800.16

Each m corresponds to four different m_0_. The confidence interval selected for each experiment was 0.95

The SD represents the discrete degree of the data. The range is the diversity between the maximum and minimum values in a list of numbers. The quartile range is the distance between upper quartile and lower quartiles. Both range and quartile range can reflect the fluctuation range and the discrete degree of the data. The root mean squared error (RMSE) is used to measure the disparity between the estimated values and the true values. The coefficient of variation (CV) is used to indicate the difference between the different indicator units. Table 4 compares the results of m_0_ estimation of the four methods using six indicators.

**Table 4:** Comparison of results of m_0_ estimation using the four methods

Indicators	ABH	S-λ	TST	UGM
Mean	2870.35	2852.16	2714.4	2849.83
SD	21.89	20.6	11.51	18.8
Range	142.72	244	67	110
quartile range	23.17	76	15	23.33
RMSE	29.93	50.36	136.08	18.71
CV	0.76%	1.77%	0.24%	0.66%

m = 3000, *π*_0_ = 0.95, *m*_0_ = *m* ∗ *π*_0_ = 2850

Table 4 showed that all the results of four methods trended to 2850. However, there was a big deviation yielded by the TST method computing the number of non-differentially genes (2714.4), i.e., the TST algorithm is less reliable for m_0_ estimation. The mean shows that the m_0_ estimated by the UGM method is the closest to the real value, which slightly better than the S-λ algorithm. In addition, the quartile range computed using ABH, UGM and S-λ method were increased. But the results of ABH and UGM method were very close to each other. What’s more, the SD, range and CV derived by the UGM method are better than both the ABH and S-λ method, which means that the discrete extent of the data calculated using the proposed method is smaller. In summary, UGM is more stable, accurate and robust. The UGM method is better than other conventional algorithms.

### Performance on real data

In order to verify the validity and accuracy of UGM, we selected the breast cancer gene chip data to further verify UGM in this paper. However, the selection of real data is random and unlimited breast cancer gene chip data, which is part of our previous research. In this paper, the gene chip data was downloaded from the NCBI\GEO database. (platforms number: GPL570; accession number: GSE31192 [5, 19]. Total RNAs were extracted from breast cancer and normal tissues. The experimental group was women with breast cancer, and the control group was women of the same age without breast cancer. Malignant epithelia and tumor-associated matrix of pregnancy-associated breast cancer (PABC) and non-PABC were isolated by laser capture micro-dissection and gene expression profile. Eventually, a total of 33 set of gene expression data composed of 20 tumors tissue and 13 normal tissues profiled by 22,283 probes were obtained.

Breast cancer gene chip data were pretreated by the RMA procedure, and all probes *P* values were computed with t-test or Satterthwaite’s approximate t-test. With FDR set at 0.05, ALSU and the UGM estimated m0 and identified the differentially expressed genes associated with breast cancer. Results were shown in Table 5.

**Table 5:** Results of identified the differentially expressed genes with the GEO database

Gene.No	Gene.symbol	UGM.adj. P.Val	ALSU.adj. P.Val	P.Value
1	CD300LG	1.49E-09	1.57E-09	2.87E-14
2	PPP1R14A	1.74E-09	1.83E-09	6.71E-14
3	PIR-FIGF	7.04E-09	7.41E-09	4.15E-13
4	SAMD5	7.04E-09	7.41E-09	5.57E-13
5	MYH11	7.04E-09	7.41E-09	6.78E-13
4281	–	4.74E-02	4.99E-02	3.91E-03
4282	TGFA	4.75E-02	5.00E-02	3.91E-03
4378	EFTUD2	4.98E-02	5.25E-02	4.20E-03
4379	TGM2	5.00E-02	5.27E-02	4.22E-03
11,318	SLC35F6	2.29E-01	2.41E-01	4.99E-02
11,319	RPS19	2.29E-01	2.41E-01	5.00E-02
54,674	EML6	1	1	1
54,675	SPG11	1	1	1

Gene. No is the ordered gene sequence. The confidence interval selected for each experiment was 0.95

The results showed that UGM algorithm and ALSU algorithm respectively yielded 4397 (8.04%) and 4282 (7.83%) differentially expressed genes. While the general t-test resulted in 11,319 (20.7%). The UGM and the ALSU were reduced by 6922 (61.2%) and 7037 (62.2%). The ALSU and the UGM methods are significantly more powerful than the general t-test (*p* = 0). What’s more, the UGM method calculating the number of differentially expressed genes were slightly higher than the result of ALSU, suggesting that the UGM method renders a more comprehensive screening results with higher efficiency and a reduced false negative rate.

Risk factors for developing breast cancer include being female, obesity, lack of physical exercise, drinking alcohol, ionizing radiation, etc. In recent years, many cancers have been recognized as inherited disease with a subset of genes mutated, including BRCA1 and BRCA2, both of which are tumor suppressor. These proteins help repair damaged DNA and, therefore, play a role in ensuring the stability of the cell’s genetic material. Specific inherited mutations in BRCA1 and BRCA2 increase the risk of female breast and ovarian cancers, and they have been associated with increased risks of several additional types of cancer. In this paper, we used the UGM algorithm to analyze the gene expression profile data of breast cancer. The results showed that BRCA1 (*P* = 0.007) and BRCA2 (*P* = 0.000129) were selected the genes susceptible to cancer (differentially expressed genes). What’s more, many genes related to BRCA1 and BRCA2 have been screened out. They are BRIP1 (*P* = 0.0000572), PTEN (*P* = 0.00399), RAD51 (*P* = 0.00389), BARD1 (*P* = 0.0344), MMP11 (*P* = 0.0256), RRM2 (*P* = 0.000823), NEK2 (*P* = 0.0000149), MKI67 (*P* = 0.000397), ITGA7 (*P* = 0.0195), CXCL5 (*P* = 0.0014).

**Fig. 2:**
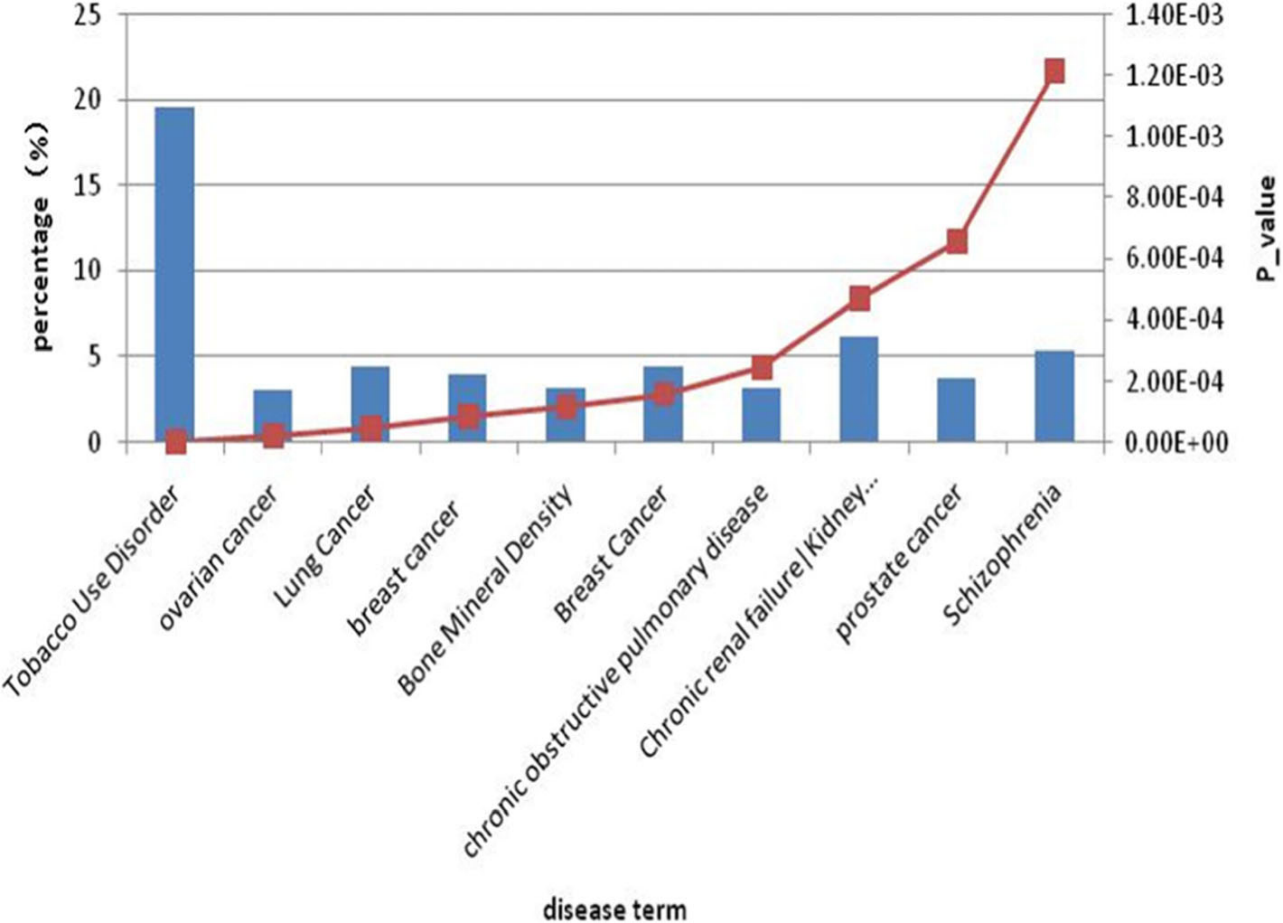
Result of gene-disease association analysis by differentially expressed genes. Note: The gene-diseases can be obtained by the differentially expressed genes list. The abscissa lists the top ten gene-disease items. The primary coordinate is the number of enriched differentially expressed genes corresponding to each genetic disease item. The secondary coordinate is enriched P value for each gene-disease

In this paper, the data we used were breast cancer gene expression profile data. we further used the DAVID Bioinformatics Resources 6.8 (https://david.ncifcrf.gov) to analyzed gene-disease association of differentially expressed genes. DAVID 6.8 allows researchers to associate sets of genes from a gene list (differentially expressed genes list) to disease phenotype, employing information from OMIM and the Genetic Association Database mapped to DAVID genes. The results showed that there were 2 terms associated with breast cancer, and 224 (8.414%) genes were enriched in disease-associated with breast cancer (p1 = 8.31E-05, p2 = 1.57E-04). The results of gene-disease association analysis by differentially expressed genes are shown in Fig. 2.

## Conclusion and discussion

In this paper, we have improved the use of *p*-value of multiple hypothesis testing in identifying disease-associated genes. The estimation results of methods were compared using simulated microarray data with mean, SD, range, quartile range, RMSE and CV as evaluation indices. The simulation results showed that the mean of non-differentially genes (m_0_) estimated by the new method was very close to the real value. The results of the UGM method and the ABH, TST method have significant differences (*P* = 0.01296, *P* = 0.0000). However, there was no significant difference between the UGM method and the S-λ method (*P* = 0.8644). These results suggested that the UGM method and S-λ method are significantly superior to the ABH and the TST methods. In addition, the SD, range, quartile range, CV and RSME of the number of non-differentially expressed genes calculated by the S-λ method were all larger than those of the UGM method and are more discrete, which is concordant with the study by Wu Jing [16]. In summary, the UGM exhibited better stability, accuracy and robustness,which was better than other conventional algorithms.

In order to verify the effectiveness of the new proposed method in screening differentially expressed genes, we used this method to calculate the gene expression profile data of breast cancer. The results displayed that the UGM method was significantly more powerful than the general t-test (*p* = 0), and has slightly larger set of differentially expressed genes than those of the ALSU, presenting lower false negative rate and higher screening efficiency. In the differentially expressed genes screened by UGM method, a bunch of well-established oncogenes and anti-oncogenes were discovered, including BRCA1, BRCA2, PTEN, BRIP1 [20], RAD51 [21], BARD1 [16, 17], MMP11 [22], RRM2 [23], NEK2 [24] et al. Furthermore, genes associated with BRCA1, BRCA2 and TP53 were also identified, such as ITGA7 [25], CXCL5 [26] etc.

Microarray technology and DNA and RNA sequencing technology produced huge amount of gene data, which has been widely used in biomedical research. The data dimension of gene expression profile is high and the sample size is small. Identifying informative candidate genes from expression profile data has become an imperative task and attracts extensive attention in the field of biology and medical statistics research. Microarrays can provide a dynamic snapshot of cell activity, but the results are not noticeable/obvious. The purpose of this paper is to provide useful answers to some of the most common practical problems in microarray data analysis, especially the multiple validation of differential expressions.

In the field of microarray data analysis, one of the critica problems of multiplicity test is to estimate the number of true null hypothesis. Traditional processes have dominated the FWER, which is the probability of type I error. When the number of genes is large, the ability to detect differentially expressed genes decreases, and the bona fide differentially expressed genes may be ignored. In actual research, identifying differentially expressed genes from expression profile data is important for gene localization, identification of biomarker and therapeutic targets and study of disease mechanism. The expected percentage of the null hypothesis that is wrongly rejected is a meaningful indicator in multiple comparisons, but not the probability of error detection. In this background, Benjamini and Hochberg [14] developed the FDR control program, which was a groundbreaking achievement. The traditional method needs tight dominate the FWER, with a conservative type I error rate dominated contra any configuration of the hypothesis tested. The FDR method keeps the error-recognition rate within the allowable range, which provides an appropriate metric for multiple tests of large-scale data. Following Benjamin and Hochberg (BH) ‘s pioneering paper, the concept of FDR has been widely used in large-scale data analysis. For the BH method, many scholars have extended on their basis and developed many excellent methods. The adaptive linear step-up (ALSU) method proposed by Benjamin et al. has been widely used in previous studies.

The key step in the ALSU process is to estimate the number of non-differentially expressed genes. However, we find that the estimation method proposed in this process is not accurate enough. Although the average of the estimated values has been very close to the true value over the course of many iterations, it is still far from the standard deviation. This introduces large amount of random errors, leading directly to inaccurate final results. In this study, we designed a new method to estimate the number of non-differentially expressed genes and proved its superiority, by using well-established microarray data.

## Data Availability

The gene chip data are available at https://www.ncbi.nlm.nih.gov/. The gene-disease association analysis is available at https://david.ncifcrf.gov. All data and materials are fully available without restriction.
